# Development of the Liverpool Adverse Drug Reaction Avoidability Assessment Tool

**DOI:** 10.1371/journal.pone.0169393

**Published:** 2017-01-03

**Authors:** Louise E. Bracken, Anthony J. Nunn, Jamie J. Kirkham, Matthew Peak, Janine Arnott, Rosalind L. Smyth, Munir Pirmohamed, Mark A. Turner

**Affiliations:** 1 Paediatric Medicines Research Unit, Institute in the Park, Alder Hey Children’s NHS Foundation Trust, Liverpool, United Kingdom; 2 Department of Women’s & Children’s Health, Institute of Translational Medicine, University of Liverpool, Liverpool, United Kingdom; 3 Department of Biostatistics, University of Liverpool, Liverpool, United Kingdom; 4 School of Health, University of Central Lancashire, Preston, United Kingdom; 5 School of Health, University of Central Lancashire, Preston, Lancashire, United Kingdom; 6 Institute of Child Health, University College London, London, United Kingdom; 7 Department of Molecular and Clinical Pharmacology, Institute of Translational Medicine, University of Liverpool, Liverpool, United Kingdom; 8 Liverpool Women’s NHS Foundation Trust, Liverpool, United Kingdom; Peking Union Medical College Hospital, CHINA

## Abstract

**Aim:**

To develop and test a new tool to assess the avoidability of adverse drug reactions that is suitable for use in paediatrics but which is also applicable to a variety of other settings.

**Methods:**

The study involved multiple phases. Preliminary work involved using the Hallas scale and a modification of the existing Hallas scale, to assess two different sets of adverse drug reaction (ADR) case reports. Phase 1 defined, modified and refined a new tool using multidisciplinary teams. Phase 2 involved the assessment of 50 ADR case reports from a prospective study of paediatric inpatients by individual assessors. Phase 3 compared assessments with the new tool for individuals and groups in comparison to the ‘gold standard’ (the avoidability outcome set by a panel of senior investigators: an experienced clinical pharmacologist, paediatrician and pharmacist).

**Main Outcome Measures:**

Inter-rater reliability (IRR), measure of disagreement and utilization of avoidability categories.

**Results:**

**Preliminary work—Pilot phase:** results for the original Hallas cases were fair and pairwise kappa scores ranged from 0.21 to 0.36. Results for the modified Hallas cases were poor, pairwise kappa scores ranged from 0.06 to 0.16.

**Phase 1:** on initial use of the new tool, agreement between the two multidisciplinary groups was found on 13/20 cases with a kappa score of 0.29 (95% CI -0.04 to 0.62).

**Phase 2:** the assessment of 50 ADR case reports by six individual reviewers yielded pairwise kappa scores ranging from poor to good 0.12 to 0.75 and percentage exact agreement (%EA) ranged from 52–90%.

**Phase 3:** Percentage exact agreement ranged from 35–70%. Overall, individuals had better agreement with the ‘gold standard’.

**Conclusion:**

Avoidability assessment is feasible but needs careful attention to methods. The Liverpool ADR avoidability assessment tool showed mixed IRR. We have developed and validated a method for assessing the avoidability of ADRs that is transparent, more objective than previous methods and that can be used by individuals or groups.

## Introduction

Adverse drug reactions (ADRs) contribute significantly to patient morbidity, mortality and hospitalisation costs. ADRs are a major patient safety issue and they can have significant consequences both for the patient and health care system [1]. A recent systematic review of ADRs in children reported incidences of ADRs in hospitalised children ranging from 0.6 to 16.8% among studies [2]. This is similar to the incidence rate in hospitalised adults of 14.7% [3]. The annual cost of drug related morbidity and mortality has been estimated in the United States at more than $136 billion and ADRs contribute significantly to these costs [4]. Davies et al. estimated that, in the UK, ADRs cost the NHS in excess of £637 million a year; this figure represents an extrapolation from a single NHS hospital to the NHS as a whole [3]. Children are considered to be particularly susceptible to ADRs [5,6].

Avoidability (or preventability, as it is also called) is an important concept in the study of ADRs [7]. The terms are often used interchangeably but for the purpose of this paper, the term avoidability has been used. A meta-analysis of avoidable ADR studies conducted by Hakkarainen et al. concluded that avoidable ADRs are a significant burden to the healthcare system and a cause of morbidity among outpatients [8]. They found that roughly half of all ADRs amongst adult inpatients (45%) and outpatients (52%) may be avoidable [8].

The study of avoidability is complex. A key factor causing this complexity is the lack of commonly accepted definitions [7]. Hakkarainen et al. listed inconsistent terminology as one of the problems; there is wide variation in the terms and definitions used (for example ADRs and adverse drug events (ADEs)) and this hinders the comparison of studies [9]. Ferner and Aronson [7] stated that there are two aspects to avoidability: whether in principle an event is avoidable in the absence of error and, if it is, whether we can in fact prevent it. There have been many attempts to devise tools or scales to help assess avoidability including from Hallas [10], Schumock and Thornton [11], Dorman et al. [12] and Olivier et al. [13]. The most commonly used scales include Hallas [10] and Schumock and Thornton [11], which are based on appropriateness of prescribing or treatment choice.

In the Adverse Drug Reactions in Children (ADRIC) programme [14], we conducted a series of studies investigating ADRs including a systematic review, an admissions study and an inpatient study. The incidence of ADRs detected at the point of admission was 2.9% with the most commonly implicated drugs being cytotoxics [6]. 17.7% of inpatients experienced at least one ADR and the most commonly implicated drugs were opioid analgesics and drugs used in general anaesthesia [15]. During the ADRIC programme we identified a need to develop a new ADR causality assessment tool following difficulties using the Naranjo tool. The Liverpool Causality Assessment Tool (LCAT) is a flow diagram designed by a multidisciplinary team (MDT) to be quick and easy to use [16]. The assessment of avoidability was central to the ADRIC study, but during the course of this programme we identified that (a) few previous studies (19/101) in the ADRIC systematic review had examined avoidability and those that had, used different methods [2]. The review concluded that the available instruments for the avoidability assessment of ADRs vary in reliability and validity [2]; and (b) difficulties were encountered during the assessment of avoidability using existing tools in the ADRIC inpatient study [15].

The Hallas scale [10] is a series of four statements linked to standards of care. Although the Hallas scale was used for the ADRIC admissions study, it appeared unsuitable for the ADRIC inpatient study as the user would need to have comprehensive knowledge of optimal treatment for all conditions represented in the cohort and the fact that guidelines are incomplete or simply do not exist. Specifically, Hallas was difficult to use in the inpatient study as treatment was often guided by tertiary paediatric specialist advice. “Present day knowledge of good medical practice” of treatments for paediatric diseases covers a vast range of information. Comprehensive awareness of the information required to assess avoidability of ADRs would require extensive reading and synthesis of information. Many paediatric conditions are rare or ultra-rare which makes information difficult to locate. In contrast, the ADRIC admissions study predominantly involved a relatively small number of common acute conditions that the research team were familiar with, or, a relatively small number of acute complications of chronic illnesses that the research team were familiar with. The Hallas scale also had a requirement to assess whether the event could have been avoided by, “an effort exceeding the obligatory demands of a case”, which was very difficult to assess consistently and objectively. In view of these issues, we decided to develop a new avoidability assessment tool. Ideally, a newly developed tool should be generalisable to a variety of different patient groups, reproducible and easy to use. The development of the new avoidability assessment tool followed a similar methodology to the development of the LCAT [16]. The aim of this paper is to report the development of the tool (termed the Liverpool avoidability assessment tool [LAAT]) and its initial evaluation.

## Methods

**Fig 1:**
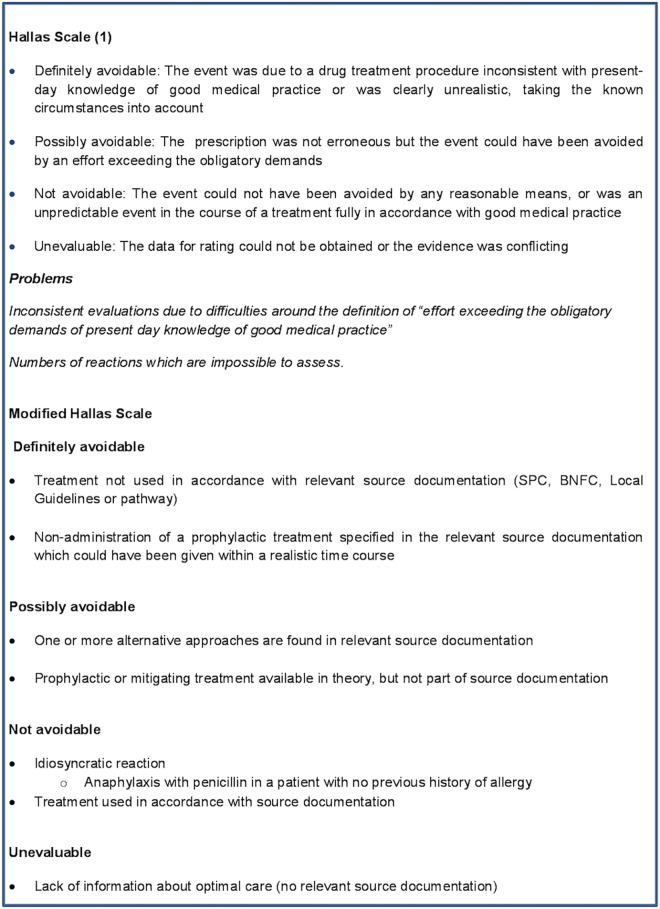
Hallas and the modified Hallas scale.

A modified version of the Hallas Scale (Fig 1) was used as the starting point for the development of the LAAT. A LAAT development team was convened and included experienced paediatricians, clinical paediatric pharmacists, a paediatric research nurse and research methodologists. The first principle that the development team agreed on was that the LAAT would be based on information available in identifiable sources that prescribers would be expected to use (rather than judgments about best practice). The intention was to keep the tool as generalisable as possible by asking if accessible management or treatment plans were available. These could be local, national or international. We recommended that only high-quality guidelines were considered. A guideline that is widely available and would be recognised as appropriate by a reasonable body of opinion for example, in the United Kingdom (UK); Scottish Intercollegiate Guidelines Network (SIGN), National Institute for Health and Care Excellence (NICE), Royal College of Paediatrics and Child Health (RCPCH) or other peer-reviewed guidelines [17]. Information sources could include the British National Formulary for children (BNF-C), Summary of Product Characteristics (SmPC) in the UK, or the local equivalent depending on where the LAAT is being used.

The second principle was, once the best available management plan had been identified, that an ADR would be unavoidable if the best available treatment or management plan was followed. Thus the steps in the LAAT fell into two types: establishing what the best available information was and then establishing whether best available information was followed. In all phases of this process, assessors were not provided with the relevant guideline and were expected to apply their awareness and understanding of the current guidelines. The development of the LAAT followed the recommendations described by Hakkarainen et al. [9]. They recommended that future studies include reliability and validity testing; take action to standardise the measurement process; provide information on the assessors in terms of training and experience in assessing avoidability; and describe how the assessments took place [9].

### Preliminary work–Pilot phase: assessment of modification of an existing tool

Three reviewers independently assessed 50 cases using a modified version of the Hallas scale (Fig 1) and 50 cases using the original Hallas scale [10]. The results were compared and inter-rater reliability (IRR) testing was carried out on both groups using kappa statistics.

It was decided that the LAAT should be converted to a flow diagram in an attempt to make it easier and more consistent to use. It was also decided that some questions needed reviewing and that this should be done by a consensus approach. We achieved consensus by agreement among peers without pre-set criteria and the consensus group was a MDT comprising a research children’s nurse, paediatrician and pharmacist.

### Phase 1: Define the tool

**Fig 2:**
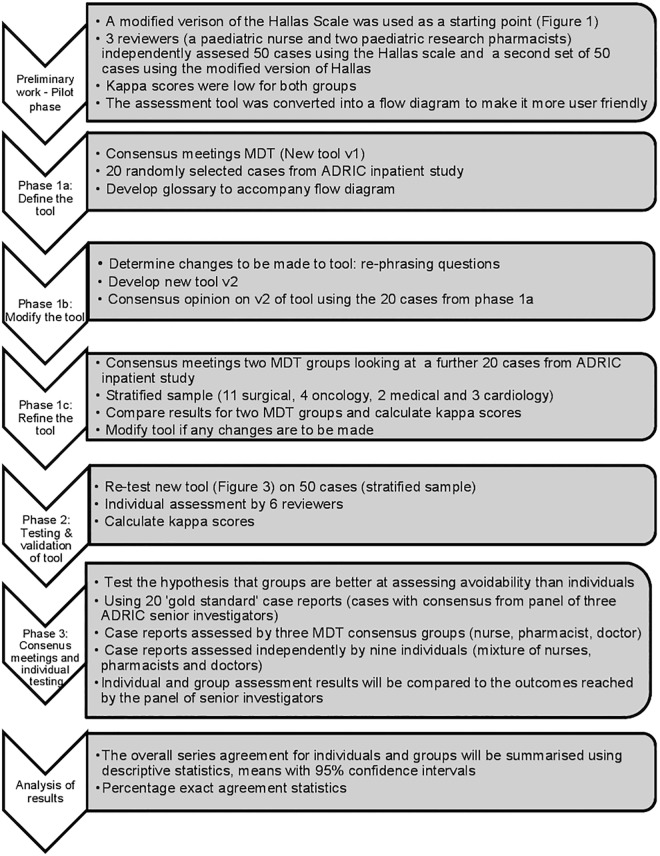
Flow chart of the development of the Liverpool ADR avoidability assesmsent tool.

The format of the new tool was a flow diagram with dichotomous responses to each question followed by a routing to the next relevant question. In Phase 1a, 20 cases were reviewed to define the tool. This was carried out by an MDT working together to discuss clinical practice and avoidability outcome and observed by a research pharmacist (Fig 2: The development of the Liverpool ADR avoidability assessment tool). Any cases which were classified as “unassessable” had the rationale recorded: due to either lack of information about the case or of available guidance. A clinical pharmacologist, who also reviewed the iterations as they moved through the various stages of development, reviewed any areas of disagreement or discrepancies. Other steps in Phase 1 are summarised in Fig 2.

### Phase 2: Testing and validation of the tool

**Fig 3:**
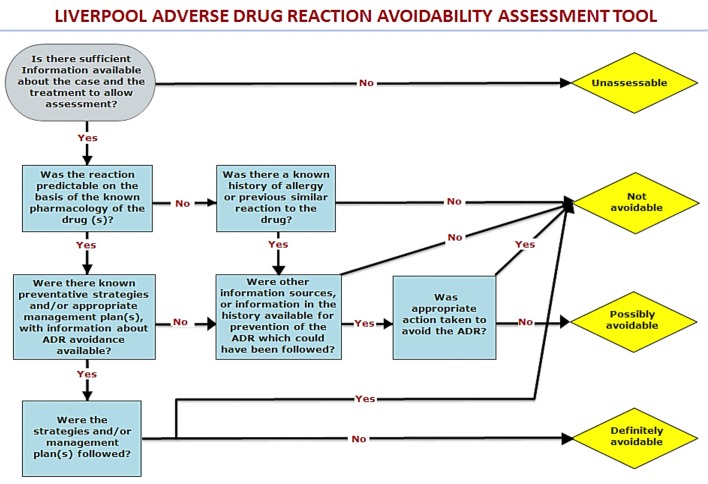
The Liverpool ADR avoidability assessment tool (LAAT).

The refined tool (Fig 3) was then tested on a further set of cases from the ADRIC inpatient study with the aim being to improve IRR. This phase involved the assessment of a further 50 cases by six individual reviewers using the newly refined tool. See the accompanying glossary and guide to the questions in the tool for further details on completing an avoidability assessment in S1 Appendix. These 50 cases were a stratified sample *(causality assessed as possible*, *probable and definite cases*: *26 surgical*, *9 oncology*, *9 medical and 6 cardiology)*. The reviewers included two nurses, two pharmacists, and two doctors. These cases were assessed in terms of pair-wise agreements between the investigators. Avoidability categories ranged from ‘unassessable’, ‘not avoidable’ ‘possibly avoidable’ and definitely avoidable’. As in the development of the LCAT cases where extreme disagreement occurred, i.e. where the avoidability assessment differed by more than one category e.g. ‘not avoidable’ and ‘definitely avoidable’ and any cases where half of the raters differed in assigning a category were identified and the questions which caused the discrepancies were reviewed [16].

Statistical analysis was carried out using IBM SPSS,Version 22 and StatsDirect Ltd. StatsDirect statistical software. http://www.statsdirect.com. The results were presented as categorical scores and inter-rater agreements were calculated using unweighted kappa scores with 95% confidence intervals (CI). Pair-wise kappa scores were compared to a global kappa score. A global kappa score measures agreement across multiple assessors [18,19]. The percentage extreme disagreement (%ED) where the avoidability scores between two raters of the same case are wider than one interval apart were calculated to measure extreme disagreement between pair-wise kappa scores. Pair-wise kappa scores were also calculated by speciality to investigate the differences between surgical, medical, oncology and cardiology cases. Kappa values were interpreted according to the guidance from Altman: poor <0.2; fair 0.21–0.4; moderate 0.41–0.6; good 0.61–0.8 and very good 0.81–1 agreement [18].

### Phase 3: Consensus methods and individual testing

None of the available formal methods were suitable for our study because the target users of this tool do not work in large groups. Accordingly we elected to use an informal method that drew on some elements of the formal methods. The tool was designed for use in real-world clinical settings. Hence the consensus method chosen should be applicable in real-world settings. The main characteristic of the informal method was small MDT groups. Each group had 3 reviewers including nurse, pharmacist and doctor with experience of prescribing, administering, or dispensing medicines for children. It was anticipated that the small groups would work informally in line with standard practice that does not include formal group processes.

Participants were assigned either to assess a selection of ADR case reports (a purposive sample of 20 ADR cases, see S1 Table) independently, or to take part in one of the three MDT consensus groups. Nine individual participants (a mixture of nurses, pharmacists and doctors) independently assessed the selected cases in their own time. Multidisciplinary groups were formed to assess the cases and reach consensus, during a 3 hour consensus group meeting. The results were compared to the ‘gold standard’—the avoidability outcome set by a panel of ADRIC senior investigators (the panel consisted a Clinical Pharmacologist, Paediatrician and Pharmacist). The extent to which individuals and groups agreed with the ‘gold standard’ using percentage exact agreement (%EA) was examined.

### Ethics statement

This study used routinely collected clinical data in an anonymized format. The Chair of the Liverpool Paediatric Research Ethics Committee informed us that this study did not require individual patient consent or review by an Ethics Committee. The participants in the study were all NHS employees who were recruited by virtue of their professional role and therefore this study did not need NHS REC approval.

## Results

### Preliminary work–Pilot phase:

The assessment of 50 ADR cases using a modified version of the Hallas scale and a second set of 50 ADR cases using the original Hallas scale by individuals (a paediatric nurse and two paediatric research pharmacists) [10]. The kappa scores for the Hallas group were fair, with pairwise kappa scores ranging from 0.21 to 0.36. The modified Hallas kappa scores were poor, with pairwise kappa scores ranging from 0.06 to 0.16.

### Phase 1

The assessment of 20 ADR cases by two MDTs showed agreement between the groups on 13/20 cases with a kappa score of 0.29 (95% CI -0.04 to 0.62). Group members commented that a mixture of professions was needed to give a full assessment of avoidability.

### Phase 2

The independent assessment of 50 ADR case reports by six individual reviewers (R1—R6) led to overall pair-wise kappa scores which ranged from poor to good (0.12 to 0.75). The assessors achieved fair inter-rater reliability with a global kappa score of 0.31. Overall percentage exact agreement (%EA) ranged from 52–90% (Table 1) and %ED ranged from 2–24%. Agreement between specialties varied. Table 2 shows the %EA and %ED between the specialties.

**Table 1:** Avoidability assessment of ADR cases from phase 2.

		**Assessor 2**						
			R1	R2	R3	R4	R5	R6
**Assessor 1**	**R1**	% EA		90%	84%	78%	60%	70%
		Kappa (95% CI)		0.75 (0.53, 0.97)	0.60 (0.42, 0.79)	0.49 (0.26, 0.72)	0.23 (0.07, 0.38)	0.34 (0.11, 0.56)
	**R2**	% EA			80%	68%	60%	60%
		Kappa (95% CI)			0.49 (0.31, 0.67)	0.26 (0.05, 0.46)	0.20 (0.03, 0.37)	0.12 (-0.09, 0.32)
	**R3**	% EA				70%	66%	64%
		Kappa (95% CI)				0.30 (0.12, 0.49)	0.32 (0.17, 0.47)	0.21 (0.03, 0.39)
	**R4**	% EA					58%	62%
		Kappa (95% CI)					0.23 (0.08, 0.38)	0.20 (-0.02, 0.42)
	**R5**	% EA						52%
		Kappa (95% CI)						0.18 (0.03, 0.33)
	**R6**	% EA						
		Kappa (95% CI)						

Kappa values were interpreted according to the guidance from Altman [18].

**Table 2:** Breakdown of percentage agreement and extreme disagreement between specialties.

Specialty	Number of ADR cases	%EA	Mean	Standard deviation	%ED	Mean	Standard deviation
**Oncology**	9	56–100%	80.2	11.16	0–22.2%	11.1	7.3
**Cardiology**	6	33–100%	59.0	17.8	0–66.7%	25.5	18.7
**Medical**	9	33–100%	69.7	20.9	0–22.2%	6.7	7.0
**Surgical**	26	50–92%	64.5	12.3	3.8–30.1%	19.1	8.8

### Phase 3

The extent to which individuals and groups agreed with the 'gold standard’ was examined using %EA. The mean agreement for individuals was 54% (SD 12.4) and 47% (SD 7.6) for the consensus groups. %EA ranged from 35–70%.

## Discussion

We have defined and validated a tool to support the assessment of the avoidability of ADRs that is based on identifiable information available to the prescriber and an evaluation of whether the management plan suggested by the best available information was followed. In addition we have compared the assessments made by groups with the assessments made by individuals. Agreement with a ‘gold standard’ was similar for individuals and groups. Given the logistical difficulties with groups, individual assessments can be used in further work. Feedback from individuals was generally positive with most participants agreeing that the tool was easy to use and that it might have potential utility in the future.

Preliminary testing of the LAAT has shown it has face validity and is easy to use. However, a number of issues were raised. These include the dependence on guidelines and variations in clinical practice. In phase 2, comparison of reviewers by specialty type highlighted that agreement was better for certain specialties, particularly oncology. This may be explained by the number of guidelines available in this area as many have detailed protocols and treatment regimens; however, the level of detail varies amongst different protocols. Also, it became apparent that despite guidelines being available for some cases, not all reviewers chose to look them up. Some cited guidelines from memory correctly or incorrectly and some clinicians used experience or prior knowledge to assess the cases. The subtlety in definitions between prevention, management and amelioration of ADRs caused confusion with reviewers. If the tool is used by experts then some of these issues may not be relevant.

Avoidability assessment is feasible but needs careful attention to methods. The LAAT showed mixed IRR when individuals made assessments; further testing was conducted to compare the use of the tool by individuals and groups. One of the limitations was the number of cases used in some of the development phases. The number of cases was limited due to practical issues and feasibility. The effect of the small number of cases used is reflected in the wide confidence intervals for the kappa scores. However, it would not have been possible to have asked healthcare professionals to assess more ADR cases as conducting avoidability assessments took a considerable amount of time. Also, there was no preset level for kappa acceptability for the development of the LAAT. It may have been useful to have assigned a minimum kappa score for agreement. As the LAAT was designed as a research tool rather than a clinical tool, the minimum value for kappa might have been set at a lower value than if the LAAT was designed as a clinical tool. For a clinical tool the minimum kappa score might be set to a minimum of 0.80 which indicates good agreement according to Altman [18] and for the LAAT as a research it might be more reasonable to set a lower kappa of 0.60 which indicates moderate agreement. A higher kappa of 0.80 may not be realistic for the LAAT given the complex nature of avoidability assessments; depending on the expertise of the reviewers conducting the assessments. In any future testing of the LAAT or in re-development of the LAAT into a clinical tool for assessing ADRs “a priori” kappa value of 0.6 could be set for the minimum level of agreement for reviewers.

The results from Phase 3 showed that individual assessments had marginally better agreement with the ‘gold standard’ evaluation than group assessments. Qualitative analysis of meeting observations may help identify reasons for this and inform the optimisation of the LAAT for use in ADR avoidability assessments. The reviewers, particularly in the groups used in Phase 3 were experienced clinicians with little or no experience with assessment of ADRs. This pragmatic selection of reviewers may have increased the variability in responses. This could be investigated in future work to look at the impact of having more experienced group members assess avoidability and compare inter-rater reliability between the different groups. External validity testing using expert groups would overcome the lack of understanding about ADRs and confusion over terminology and is therefore likely to improve the tool’s IRR. In addition, the provision of training or additional instruction about the use of the tool might help to improve agreement. A pilot study, randomised controlled trial (RCT) was conducted to test whether an e-learning tool which was developed to provide training in the use of the Liverpool causality assessment tool (LCAT) improved the ability of medical trainees to assign ADR causality using a series of reference ADR cases [20].

Although the LAAT was predominantly designed as a research tool it may have an application in other settings. It could potentially be used for organisational change. Inviting members of the drug and therapeutics committee and senior clinicians with experience in the regulatory field to assess the avoidability of a selection of ADR case reports using the LAAT, may help to identify prevention strategies. A thorough exploration of avoidable ADR cases could inform the development of practical interventions that can be translated into clinical practice. In conclusion we have developed and validated a method for assessing the avoidability of ADRs that is transparent, more objective than previous methods and that can be used by individuals or groups.

## Supporting Information

S1 Appendix Liverpool ADR avoidability assessment tool glossary. (DOCX)

S1 Table Phase 3—Avoidability category assignments of individual reviewers and consensus groups—a comparison to the ‘gold standard’. (PDF)
