# Supplementary material for: Development of the Liverpool Adverse Drug Reaction Avoidability Assessment Tool
Source: PLoS One. 2017 Jan 3;12(1):e0169393. doi: 10.1371/journal.pone.0169393 (PMC5207751; doi:10.1371/journal.pone.0169393)
Supplement: S1 Appendix — (DOCX) [file pone.0169393.s001.docx]

**Liverpool ADR avoidability assessment too (LAAT) glossary**

- **Known preventative strategies:** prophylactic or concomitant medicines or any necessary monitoring.
- **Appropriate management plan(s):** a plan that would be recognised as appropriate by a reasonable body of opinion. This could refer to **any** local, national or international guideline that could be available to the prescriber e.g. hospital guidelines, National Institute for Health and Care Excellence (NICE), Scottish Intercollegiate Guidelines Network (SIGN), British thoracic society (BTS), the British society for paediatric and adolescent rheumatology (BSPAR) or World health organisation (WHO) or a ‘personalised management plan’.

For example in the case of post-operative nausea and vomiting; examples of appropriate management plans could include: Alder Hey Children's NHS Trust guideline on post-operative nausea and vomiting or the association of paediatric anaesthetists of Great Britain and Ireland (APA) guideline on the prevention of post-operative vomiting in children.

- **Information about the ADR and its avoidance:** does the management plan mention any preventative measures to be taken to avoid the ADR including medicines to be given prophylactically or concomitantly or any necessary monitoring etc. (electrolytes, FBC or BP)? A management plan may or may not contain information regarding prevention of ADRs but more often than not they contain no information regarding the prevention of ADRs.
- **Other information sources:** examples include the BNFC (British National Formulary for children), SmPC (Summary of Product Characteristics), advice from colleagues, history from the parents/patients or information from a journal article etc. (if the prescriber could reasonably be expected to use these sources)
- **Unassessable:** the case could not be assessed due to lack of information about the case and/or treatment; or due to conflicting information.
- **Not avoidable:** the ADR was unavoidable based on the information available at the time of the reaction. There are four scenarios which lead to an ADR being categorised as “not avoidable”

1. If the reaction was unpredictable and there was no known history of previous similar reaction or allergy to the drug.
2. If there was an appropriate management plan with information about the ADR and its avoidance and it was followed.
3. If there was no appropriate management plan, with information about the ADR and its avoidance available, there were no other information sources available to consult and there was no information in the history available for prevention of the ADR.
4. If there was no appropriate management plan, with information about the ADR and its avoidance available but there were other information sources available to consult or information in the history available for prevention of the ADR and appropriate action was taken to avoid the ADR.

- **Possibly avoidable:** there was no appropriate management plan available to follow but there were other information sources or information in the history available to prevent the ADR and these were not followed.
- **Definitely avoidable:** there were known preventative strategies or an appropriate management plan was available with information about the avoidance of the ADR but the strategies and or management plan were not followed.

**Guide to questions in the avoidability tool**

**Is there sufficient information available about the case and the treatment to allow assessment?**

If the answer is ‘yes’ there is sufficient information available about the case and the treatment then the assessor can proceed to the next question if the answer is ‘no’ either due to lack of information or conflicting information the case becomes ‘unassessable’ (this category may not be assigned until the case has been reviewed and guideline/information sought).

**Was the reaction predictable on the basis of the known pharmacology of the drug(s)?**

This question relates to whether the ADR is predictable on the basis of known pharmacology as there is lots of ‘unknown’ pharmacology. If the answer is ‘no’ then you proceed to the question asking if there was a known history of a previous similar reaction. If the answer is ‘yes’ then you proceed down the left hand side of the flow diagram where you are asked questions regarding availability of appropriate management plans and if they were followed.

**Was there a known history of allergy or previous similar reaction to the drug?**

The purpose of this question is to establish if the patient has experienced a similar reaction in the past and by answering ‘no’ to the question takes you to ‘not avoidable’ as for unpredictable reactions where the patient has no previous history of it occurring the reaction could not have been prevented on this occasion. In theory this reaction could be avoided in the future.

**Were other information sources, or information in the history available for prevention of the ADR which could have been followed?**

This is an important question to establish if there was something else which could have been done to avoid the ADR either by consulting a more senior clinician for advice or looking in another reference source; examples include but are not limited to BNFC (British National Formulary for children), SmPC (Summary of Product Characteristics), consulting the parents or conducting a quick search for journal article etc. if the answer is ‘no’ to this question then the reaction is categorised as ‘not avoidable’ if the answer is ‘yes’ you proceed to the next question.

**Was appropriate action taken to avoid the ADR?**

This question allows the reaction to be categorised as ‘not avoidable’ if appropriate action was taken to avoid the ADR but it occurred anyway and for cases where other information sources were available but the appropriate action was not taken i.e. answering ‘no’ to the question categorises the ADR as ‘possibly avoidable’.

**Were there known preventative strategies and/or appropriate management plan(s), with information about the ADR and its avoidance, available?**

This question is designed to establish if there was an appropriate treatment guideline available. This could include **any** local, national or international guideline available to the clinical team when the child was seen e.g. hospital guidelines, NICE (National Institute for Health and Clinical Excellence), SIGN (Scottish Intercollegiate Guidelines Network), BTS (British thoracic society), WHO (World health organisation). If there was information available on the management of the condition but the guidance makes no reference to the ADR or its prevention then by answering ‘no’ to the question you are directed to answer the question about whether other information sources were available. This allows for the application of other measures. If the answer is ‘yes’ to this question you proceed to the next question below.

**Were the strategies and/or management plan(s) followed?**

If there was an appropriate management plan available and it contained information about the avoidance of the ADR but it was not followed this would mean by answering ‘no’ to this question categorises the ADR as ‘definitely avoidable’ if the answer is ‘yes’ the drug(s) was used in accordance with the management plan then the ADR is categorised as ‘not avoidable’.
