# Supplementary material for: Development of the Liverpool Adverse Drug Reaction Avoidability Assessment Tool
Source: PLoS One. 2017 Jan 3;12(1):e0169393. doi: 10.1371/journal.pone.0169393 (PMC5207751; doi:10.1371/journal.pone.0169393)
Supplement: S1 Table — (PDF) [file pone.0169393.s002.pdf]

Supplementary Table 1. Phase 3 - Avoidability category assignments of individual reviewers and consensus groups - a comparison to the 'gold standard'

ADR Type	Suspected Drug(s)	Causality	Gold Standard Avoidability Outcome	N1	N2	N3	D1	D2	D3	P1	P2	P3	Consensus Group 1	Consensus Group 2	Consensus Group 3
**Vomiting**	Cytarabine<br>Ifosfamide<br>Methotrexate<br>Etoposide phosphate	Definite	Definitely Avoidable	NA	NA	NA	DA	DA	DA	DA	NA	NA	NA	NA	NA
**Procedural vomiting**	Morphine Sulphate<br>Propofol<br>Nitrous oxide<br>Sevoflurane<br>Fentanyl citrate	Probable	Definitely Avoidable	NA	PA	PA	DA	DA	DA	NA	DA	DA	DA	DA	DA
**Somnolence**	Codeine Phosphate<br>Chlorphenamine	Probable	Definitely Avoidable	NA	PA	NA	NA	PA	PA	NA	NA	DA	NA	NA	PA
**Immunosuppressant drug level increased**	Tacrolimus<br>Clarithromycin	Probable	Definitely Avoidable	NA	NA	PA	DA	PA	DA	PA	PA	DA	DA	DA	DA
**Constipation**	Fentanyl citrate<br>Codeine phosphate	Probable	Definitely Avoidable	NA	DA	NA	DA	PA	DA	NA	PA	DA	DA	DA	NA
**Hallucination**	Morphine Sulphate	Probable	Not avoidable	NA	NA	NA	NA	UA	NA	NA	NA	NA	NA	DA	UN
**Cardiac failure**	Bisoprolol fumarate<br>Carvedilol	Definite	Possibly avoidable	PA	DA	NA	NA	NA	NA	NA	NA	PA	NA	NA	NA
**Respiratory depression**	Morphine sulphate	Definite	Possibly avoidable	PA	NA	PA	DA	PA	PA	PA	NA	NA	DA	DA	PA
**Flushing**	Dexamethasone	Definite	Not avoidable	NA	NA	UN	NA	NA	NA	PA	NA	NA	PA	PA	NA

ADR Type	Suspected Drug(s)	Causality	Gold standard avoidability outcome	N1	N2	N3	D1	D2	D3	P1	P2	P3	Consensus group 1	Consensus group 2	Consensus group 3
**Pruritus**	Fentanyl & Levobupivicaine	Probable	Not avoidable	NA	NA	NA	NA	NA	NA	NA	NA	NA	DA	DA	DA
**Visual disturbance**	Morphine sulphate	Probable	Not avoidable	NA	NA	NA	UA	NA	NA	NA	NA	NA	NA	NA	NA
**Diarrhoea + Excoriation**	Cefotaxime Flucloxacillin Clindamycin	Probable	Not avoidable	NA	PA	NA	NA	PA	NA	PA	PA	PA	DA	DA	PA
**Hypocalcaemia + Hypophosphataemia**	Pamidronate	Probable	Not avoidable	NA	UN	PA	NA	PA	PA	NA	PA	DA	PA	NA	NA
**Haematemesis**	Aspirin	Probable	Not avoidable	NA	PA	UN	PA	PA	DA	PA	NA	NA	UA	PA	NA
**Hypertension**	Prednisolone	Probable	Not avoidable	NA	NA	NA	NA	NA	NA	PA	NA	NA	DA	PA	DA
**Infusion associated reaction**	Rituximab	Probable	Not avoidable	NA	NA	DA	NA	NA	NA	NA	NA	DA	NA	NA	NA
**Urinary retention**	Morphine	Definite	Not avoidable	NA	NA	NA	NA	NA	NA	PA	NA	NA	NA	NA	NA
**Clostridium difficile colitis**	Teicoplanin Piperacillin, tazobactam Gentamicin	Definite	Not avoidable	NA	NA	NA	NA	PA	PA	NA	DA	DA	NA	UA	NA
**Stomatitis**	Clofarabine Cyclophosphamide Etoposide phosphate	Probable	Not avoidable	NA	DA	NA	DA	PA	NA	PA	NA	DA	NA	NA	NA
**Hypoglycaemia**	Insulin detemir	Probable	Possibly avoidable	NA	DA	NA	NA	NA	NA	NA	NA	DA	NA	NA	DA

Key

Possibly avoidable - PA

Definitely avoidable - DA

Not avoidable -NA

Unassessable - UN

Nurse 1 -N1

Nurse 2 -N2

Nurse 3 - N3

Doctor 1 - D1

Doctor 2 - D2

Doctor 3 - D3

Pharmacist -P1

Pharmacist - P2

Pharmacist 3 - P3
